# Optimization of ionic strength of nutrient solution for enhanced hydroponic watermelon yield and quality in greenhouse

**DOI:** 10.1038/s41598-025-07250-9

**Published:** 2025-07-17

**Authors:** Md Mukhtar Hossain, Yuki Shibasaki, Rina Nakao, Kazuki Nitano, Fumiyuki Goto

**Affiliations:** 1 The United Graduate School of Agricultural Sciences, Kagoshima University, Kagoshima, 890-0065 Japan; 2 Faculty of Agriculture, Rajshahi University, Rajshahi, 6205 Bangladesh; 3 Faculty of Agriculture, Saga University, Saga, 840-8502 Japan

**Keywords:** Watermelon, Nutrient solution, Solution strength, Nutrient management, Hydroponics, Greenhouse cultivation, Physiology, Plant sciences

## Abstract

Optimizing nutrient solution concentration (NSC) in hydroponic systems is crucial for enhancing nutrient efficiency and maximizing crop growth, yield, and quality. This study evaluated the effects of different NSCs on physiological responses, growth parameters, yield, and quality of watermelon (*Citrullus lanatus*) in a vertical farming system within a greenhouse in Japan from May to August 2024. Four NSCs were tested: Enshi Shoho (control), Formula-1, Formula-2, and Formula-3. These formulations were based on nutrient absorption rates determined by high-pressure ion chromatography (HPIC) in a preliminary study. The results demonstrated significant variations in vegetative growth, physiological responses, and yield attributes among treatments. Formula-1, with a reduced NSC, exhibited increased leaf area, chlorophyll content, photosynthetic rate, stomatal conductance, transpiration rate, plant fresh weight, and dry weight. It also produced larger ovaries and fruits, increasing yield by 14.8% compared to the control. Additionally, Formula-1 showed higher Brix (12.00), lycopene content, and flesh thickness (154.26 mm), indicating early maturity due to higher sugar and lower acid content. Performance ranking was Formula-1 > Formula-3 > Control > Formula-2. Thus, Formula-1 was identified as the optimal NSC for maximizing watermelon growth, physiological efficiency, yield, and quality in hydroponic cultivation.

## Introduction

Watermelon (*Citrullus lanatus*) is a major, high-production fruit vegetable in Japan with yearly production following that of cucumbers and tomatoes^1^. Total global production reached 118 million metric tons in 2022^2^. It stands out as a popular and economically significant fruit throughout the world, known for its refreshing taste and nutritional value^3,4^. Red fleshed watermelons are an excellent source of the phytochemical lycopene^5^. Although watermelon is cultivated in field conditions, in Japan it is one of the major vegetables subject to both in greenhouse and field conditions.

A vertical farming system inside greenhouse assures higher production through maximum utilization of land and space. Generally, hydroponic cultivation is more popular in the vertical farming system in the greenhouse. It has emerged as an excellent alternative system for crop production without soil^6^. In hydroponics, nutrients are supplied as ions in the nutrient solution^7,8^. Several formulations of essential macro- and micronutrients have been developed to enhance nutrient uptake and plant growth^7,9–11^. That’s because the nutrient solution is the only source of mineral nutrients in hydroponically grown plants. The composition of culture solutions is crucial for increasing the efficiency of nutrients and maximizing the growth, yield and quality of crops^12,42,43^. Very low and very high concentrations inhibit plant growth as well as reducing overall yield^13,41^. Optimized nutrient solutions for many crops have been developed for hydroponics, but there are no optimized nutrient solution concentrations (NSC) for watermelon, because watermelon is typically grown in fields.

NSC tailored to the specific needs of the watermelon plant can significantly impact its growth, development and nutritional composition. Numerous studies have shown that NSC impacts the growth, yield and quality of crops such as strawberries [40,48], lettuce^14^, tomato^15–17,45,51]^, cucumber^18,19^, bean^20^, onion^21^, melon [50]. The chlorophyll index and sugar percentage and lycopene content of fruit largely depends on NSC^15,17^,41]. Understanding the optimum NSC of watermelon plants within controlled environment in greenhouse is crucial for maximizing yield and quality of watermelon^22^. The Enshi shoho Nutrient formula^23^ is widely used in Japan for vegetable crop cultivation^24^. This formula is common for all types of vegetables but no studies in watermelon plants have been carried out by modifying this formula based on the actual requirements for watermelon plants.

Hence, the main objective of this study was to determine the optimal NSC for producing high quality watermelon. To this end, we first investigated the nutrient absorption characteristics of watermelon. Based on these findings, three different formulations were designed. Finally, we compared the physiological responses, growth parameters, and yield of watermelon across the different formulations with the control, all within the controlled environment inside greenhouse.

## Materials and methods

### Plant materials and cultivation conditions

Small fruited watermelon (*Citrullus lanatus*) cultivar; Hitorijime 7 were selected for this experiment. The experiment was conducted in hydroponic system inside greenhouse, Japan, located at 33° 16′ 50.7″ N 130° 18′ 09.9″ E, at an altitude of 4.7 m during the period from May to August 2024. The climatic parameters including temperature, relative humidity and daily light integral (DLI) both the inside and outside of the greenhouse during the experimental period are presented in Table 1. Seeds were sown in a black plastic seedling pot (length 5.5 cm, upper portion 6 cm and lower portion 4 cm in diameter). After sowing seeds, the seedling pots were kept at 24–30 °C and provided with a 12-h lighting period with light intensity of 1500 µmol/m^2^/sec. 20 days after sowing seeds, the seedlings were transplanted in the greenhouse.

**Table 1:** The climatic parameters for the experimental area during the experimental period were calculated as monthly mean values, both inside and outside the greenhouse.

Month	Inside the greenhouse^a^				Outside the greenhouse^b^			
	T (°C) Maximum minimum		RH (%)	DLI (mol m⁻^2^ d⁻^1^)	T (°C) Maximum minimum		RH (%)	DLI (mol m⁻^2^ d⁻^1^)
May	25.1	16.0	71	12.5	24.8	15.6	72	18.2
June	30.2	21.5	79	14.2	27.0	19.3	79	17.4
July	38.5	24.9	80	15.5	33.5	25.0	78	18.5
August	39.1	25.5	79	13.6	33.6	25.7	79	17.2

^a^The average temperature and relative humidity were measured by using a wireless thermos recorder, RTR503B, serial no.060B7A28 (T & D corporation, Japan) & DLI was measured using an OptoLeaf D-Meter RYO-470 M (Taisei Fine Chemical, Asahi, Japan).

^b^Japan Meteorological Agency|Climate of Japan, 2024.

### Preparation and management of nutrient solution

In this study, we used 4 different nutrient formulas including Enshi shoho solution as a control^23^. The remaining three formulas were designed by us based on the results of our preliminary experiments results. The nutrient absorption rate by watermelon plants was determined based on the absorption rate measured using high pressure ion chromatography (HPIC). In this experiment seedlings of the same size were transplanted in three litter containers with the same cultivate solution: Enshi shoho^23^ in DFT system. We added only water when the cultivated solution was decreased through absorption by the plants and maintained the same level during the experimental period. The sample was collected at 3 day intervals and diluted 50 times with ultrapure water to a total volume of 2.0 ml. The diluted sample was mixed by inversion 10 times, drawn up with a 1.0 mL syringe, and then injected into a 1.5 mL vial through a membrane filter (Shimadzu GLC TORAST Disc) with a diameter of 13 mm and a pore size of 0.45 μm. The prepared samples were placed in the DIONEX INTEGRION HPIC (ThermoFisher Scientific, Waltham, MA, USA) machine, and using DIONEX AS-AP Autosampler both anions and cations were measured and analyzed according to the machine’s manual. After getting the HPIC result we could easily understand the nutrient preferences of watermelon plants. By considering this we made the formulas; the EC were 2.55, 2.15, 2.95 and 3.35 ds m^−1^ in control, Formula-1, Formula-2 and Formula-3 respectively (Table 2). The pH was maintained from 5.80 to 6.20 during the cultivation period using HCl and KOH.

**Table 2:** The ionic strength of nutrient solution used in these experiments.

Component	Amount of nutrientfor 1 Litter solution			
	Control	Formula-1	Formula-2	Formula-3
KNO3	80.8 g/L	100.5 g/L	161.6 g/L	121.2 g/L
Ca (NO3)_2_ ·4H_2_O	94.4 g/L	50.3 g/L	47.2 g/L	94.4 g/L
Mg SO4·7H_2_O	49.2 g/L	36.9 g/L	49.2 g/L	49.2 g/L
NH4·H_2_PO4	15.2 g/L	25.5 g/L	15.2 g/L	30.4 g/L
Fe-EDTA	2.4 g/200 ml	1.8 g/200 ml	2.4 g/200 ml	2.4 g/200 ml
H3BO3	0.3 g/200 ml	0.23 g/200 ml	0.3 g/200 ml	0.3 g/200 ml
MnSO4(4–7) H_2_O	0.2 g/200 ml	0.15 g/200 ml	0.2 g/200 ml	0.2 g/200 ml
ZnSO4·7H_2_O	0.22 g/100 ml	0.15 g/100 ml	0.22 g/100 ml	0.22 g/100 ml
CuSO4·5H_2_O	0.05 g/100 ml	0.03 g/100 ml	0.05 g/100 ml	0.05 g/100 ml
Na2MoO4	0.02 g/100 ml	0.01 g/100 ml	0.02 g/100 ml	0.02/100 ml

Control (Full strength of Enshi shoho), and Formula 1, Formula 2, and Formula 3 prepared by us based on the nutrients absorption rate by watermelon plant which was observed by ion chromatography (HPIC) reports in our previous study.

The EC were 2.55, 2.15, 2.95 and 3.35 ds m^−1^ in control, Formula-1, Formula-2 and Formula-3 respectively. The pH was maintained from 5.80 to 6.20 during the cultivation period using HCl and KOH.

### Experiment layout and design

**Fig. 1:**
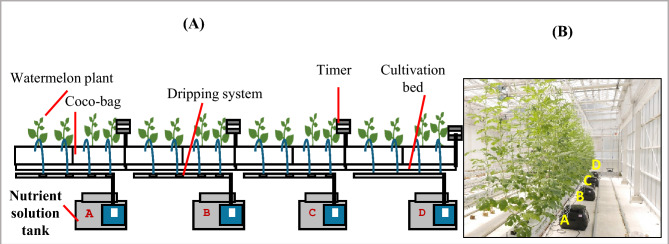
System diagram for the application of nutrient solution (**A**). The cultivate condition inside greenhouse with nutrient supply (**B**). Four nutrient solution were placed into the four different tank and four different pump were used to supply the nutrient solution to plant, pump were connected to the timer, timer on 9.00–9.15, 12–12.15 and 15.00–15.15 transplanting to pollination, 9.00–9.20, 12.00–12.20 and 15.00–15.20 from fruit setting to harvesting everyday. Here, A = Control, B = Formula-1, C = Formula-2, D = Formula-3.

A completely randomized design (CRD) was employed to evaluate the effects of different nutrient solution concentrations (NSC) on watermelon growth and quality. Four different NSC treatments; control (Enshi shoho), Formula-1, Formula-2, Formula-3 were used in this experiment (Table 2). The watermelon plants were grown vertically inside the greenhouse. The seedlings in 20 days of the same size were transplanted in coco bags (ToyoTane Co. Ltd, Aichi, Japan, length 90 cm, width 18 cm, height 5 cm and weight was 3.5 kg) Fig. 1. Each coco-bag contained two plants, and each treatment contained six plants. The plant-to-plant distance was 40 cm, and the row length was 3.2 m. We used four different black tanks (hundred liter) and four different nutrient formulas were placed into these tanks separately. Four separate water pumps (mighty pump, PSP-100S, China) of the same type were used to supply the nutrient solution to the plants in a dripping system. The pumps were connected to the timer for automatic nutrient supply Fig. 1. The timers were on three times a day 9.00–9.15, 12.00–12.15 and 15.00–15.15 from transplanting to pollination and 9.00–9.20, 12.00 to 12.20 and 15.00 to 15.20 from pollination to harvest. In the drip irrigation system, each drip hose releases nutrient solution at a rate of 22.5 mL per minute. With four drip hoses per coco bag, each bag receives a total of 90 mL per minute. When the ninth leaf was produced, the top was cut to create branches, maintaining four branches per plant. The plants flowered 20 to 24 days after transplanting and were hand-pollinated to set the fruits. Two fruits were maintained on each plant. All fruits were harvested 36 days after pollination on 1 August 2024.

### Plant sampling, measurements and analysis

The chlorophyll content of the 9th leaf from the apical tip was determined using a chlorophyll meter (SPAD-502 Plus, Konica Minolta, Ltd., Japan) at 15, 30 and 45 days after planting (DAP). The measurements of photosynthetic gas exchanges were taken on the upper surface of leaves at 10.00–12.00 h. Net photosynthesis (µmol/m^2^/sec), transpiration rates and stomatal conductance were measured by an infrared gas exchange analyzer (MIC-100 system, M20 19F1023, Masa Int., Kyoto, Japan). Measurement was taken in 7th leaf from the top and six individual plants from each treatment at 15, 30 and 45 DAT from 10.00 to 12.00 h. CO_2_ concentration measured in the chamber and measurements of variation are in the range of 10 ppm from 380 to 370 ppm. Measurements can be made every 0.1 s with 0.1 ppm resolution for CO_2_ concentration. The average temperature and relative humidity by using a wireless thermos recorder, RTR503B, serial no.060B7A28 (T & D corporation, Japan) during the growing season. Phenotypic data on various growth parameters and fruit characteristics, including plant height, were measured with a metric ruler from 15 days interval from plant to harvesting stage. The fruit diameter at10, 20 and 30 DAP were measured by using Digi Matic Caliper (Mitutoyo, Japan). The number of male and female flowers (from flowering to 20 days later) were counted and fruit weights were measured by using spring indicator scale at 10, 20 and 30 DAP. At harvest, the fresh weight of shoot and dry weight of shoot were measured. Color (L*, a*, b*) using (Minolta Chromameter (CR-10), sugar and acid contents were measured by using a Brix-Acidity Meter (PAL-BX or ACID F5, Atago, Japan). Juice PH was measured by Personal P^H^/ORP meter (PH72, Yokogawa Electric Corporation, Tokyo).

### Statistical analysis

Data was analyzed using the JMP Pro statistical package (SAS Institute, Cary, NC, USA) software; version no. 17.2.0. Significant differences among treatments were determined by analysis of variance (ANOVA). Tukey’s multiple range test was used to evaluate treatment effects and conduct comparisons, while the least significant difference (LSD) test at *p* ≤ 0.05 was used.

## Results

### Determination of nutrient absorption rate from preliminary research results

**Fig. 2:**
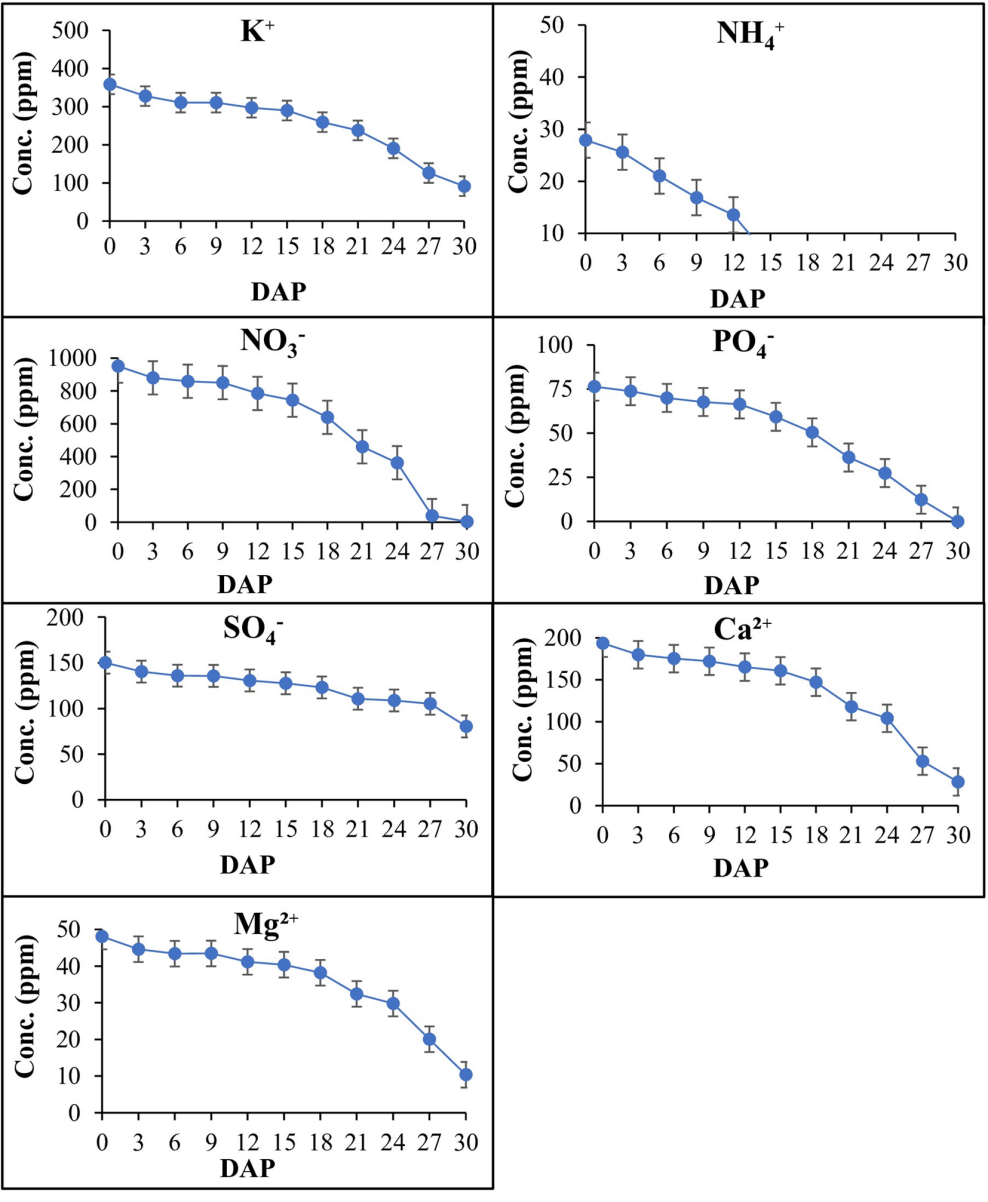
The application and remaining amount of nutrient by absorbing the watermelon plants. The curves indicated that the nutrient remains amount at different days after planting (DAP). The absorption rate was observed by high pressure ion chromatography (HPIC) at 3 days interval. *n* = 6 and Bars indicates Standard Error (S.E).

First, a preliminary experiment was conducted to understand the nutrient absorption rate for making the formula. In this experiment, deep flow technique (DFT) of hydroponic were used according to the methods of Goto et al.^25^, which is easy to understand the tendency of uptake from roots. Figure 2 showed the application and absorption rate of nutrient solutions. These results demonstrated that NH4^+^, PO4^−^ and NO3^−^ were absorbed faster than any other nutrients. Within 18 days the watermelon plant absorbed all NH4^+^ and within 30 days all NO3^+^ and PO4^−^, while the absorption rate of other nutrients was low. After 30 days we observed K^+^, SO4^−^, Ca^2+^ and Mg^2+^ remained in cultivate solution that was applied during starting this experiment. At the end of preliminary experiments, we found that watermelons absorb N (NO3^−^, NH4^+^), and P (PO4^−^) faster than another element. The absorption rate of nutrient solution was estimated from the daily changes of the concentration of culture solution (Fig. 2). As we know from our preliminary experiment, which element absorbs more, and which elements absorb less, this theme was very helpful for making the formula that was used in our main experiment.

### Consideration of preliminary research report for making different nutrient formulae

The Enshi shoho nutrition solution served as the control, while three additional treatments were designed based on the results of the preliminary experiment (Fig. 2). We developed three formulas: one with low NSC and two with high NSC compared to the control formula. The EC value for Enshi shoho (control) were 2.55 ds m^−1^ and Formula-1, Formula-2, and Formula-3 were 2.15, 2.95 and 3.35 ds m^−1^ respectively. In both low and high NSC conditions, the concentrations of N and P were utilized at elevated levels relative to other nutrients because our preliminary experiment showed watermelon prefer nitrogen (NO3^−^ & NH4^+^) and phosphorous fertilizer, and during making these three formulae we considered this effect. During designing the formula-1 the nutrients NO3^−^, NH4^+^, and PO4^−^ were elevated compared to the control, and other nutrients were reduced compared to control, because we focused on low concentration with actual nutrients requirement for watermelon plants.

The rationale for utilizing low NSC is that the experiment was conducted in a hydroponic system, and the plants were transplanted into coco bags during the summer season. The coco bags frequently dried, necessitating prolonged pump operation, which resulted in the delivery of a greater volume of nutrient solution compared to the winter season. Conversely, elevated NSC levels were utilized as our preliminary investigations indicated that within 30 days, the plant assimilated all the initially applied amounts of N and P (Fig. 2). While producing high NSC, we exclusively increased the quantities of nitrogen and phosphorus beyond those of Enshi shoho. From the result of the preliminary experiment, Formula-1, Formula-2 and Formula-3 were selected. Formula-1 was the low NSC compared to other treatments, but the concentrations of N and P were utilized at elevated levels relative to other nutrients used in Formula-1. Formula-2 and Formula-3 were high NSC with high levels of N and P. The difference between Formula-2 and Formula-3 was that in Formula-2 high K doses were used compared to Formula-3. The Enhanced K dose increases the yield and quality of watermelons^26^ and Formula-3 used high PO4^−^ and NH4^+^ compared to other treatments. Table 2 shows the ionic strength of the 3 formulations with control formulations.

### Effect of NSCs on physiological characters

Investigating chlorophyll content and photosynthesis is essential because there is a strong correlation between chlorophyll content, photosynthesis efficiency, and plant growth [44,49]. In the different growth stages, the leaf chlorophyll index content varies with different NSC^20^. We measured chlorophyll content at 15, 30 and 45DAP and found the highest value in Fornula-1 with significant differences among other treatments (Table 3). The photosynthesis rate is presented in (Table 3) and shows the increased rate of photosynthesis with decreased nutrient level in Formula-1. The stomatal conductance and transpiration rate was varied with different NSC and found the highest value in Formula-1 (low NSC) compared to control and all other NSC (Table 3). Formula-1 outperformed compared to control and all other treatments in case of chlorophyll content, photosynthesis rate, stomatal conductance and transpiration rate (Table 3).

**Table 3:** Effects of different formula on chlorophyll index (SPAD), photosynthetic rate (*Pn*), Transpiration rate (*E*), and Stomatal conductance (*Gs*) in three different stages at 15, 30 and 45 days after planting.

Treatments	Chlorophyll index (SPAD)			Pn (µmol CO2 m^−2^ s^−1^)			E (mmol H_2_O m^−2^ s^−1^)			Gs (mmol m⁻^2^ s^−1^)		
	DAP			DAP			DAP			DAP		
	15	30	45	15	30	45	15	30	45	15	30	45
Control	51.8	52.7	50.9	17.3	23.8	22.1	6.83	9.44	8.8	300.7	565.5	488.4
Formula1	64.5	64.4	62.5	20.8	27.6	29.5	8.25	10.96	11.7	432.6	763.4	871.4
Formula2	52.9	55.2	61.9	17.2	27.1	26.1	6.83	10.76	10.4	296.5	735.5	682.2
Formula3	63.2	56.9	49.9	20.5	20.5	22.7	8.13	9.83	9.0	420.2	613.6	516.2
F-test	**	**	**	**	**	**	**	ns	**	**	**	**
C.V (%)	6.10	7.0	8.7	4.5	6.1	5.5	10.5	5.0	5.5	11.0	9.8	6.1

**Indicated significant differences were found among the treatment and ns indicated non-significant differences at *p* < 0.05; Turkey’s HSD test, *n* = 6.

### Effect of NSCs on the growth and morphological characters

The growth of watermelon plants was significantly influenced by different rates of NSC. The plant height, number of female flowers per plant, leaf area, stem diameter, fresh weight and dry weight of shoots were found different under four different treatments. The low NSCs in Formula-1 found higher growth performance among all growth parameters except fresh weight and dry weight of shoot compared to all other treatments and created significant differences (Table 4). The Formula-1 showed highest plant height (598.33 cm), while control, Formula-2 and Formula-3 plant height were 562.83 cm, 497.16 cm and 527.33 cm respectively.

**Table 4:** Different growth and yield contributing characteristics of watermelon influences by different formula.

Treatments	Plant height (cm)	No. of female flowers	Leaf area (cm^2^)	Stem diameter (mm)	Fresh weight of shoot (kg)	Dry weight of shoot (g)	Yield plant^−1^ (kg)	Yield increased (%)
Control	562.83b	20b	156.07b	13.72ab	2.4ab	199.1bc	3.58b	100
Formula-1	598.33a	25a	209.88a	14.17a	1.8b	187.71c	4.11a	114.8
Formula-2	497.17d	20b	158.52b	12.65b	2.2ab	262.34b	3.54b	98.88
Formula-3	527.33c	19b	186.34ab	12.09b	2.6a	294.31a	3.80ab	106.14

Different letters indicate significance difference (*p* < 0.05; Turkey’s HSD test), *n* = 6.

**Fig. 3:**
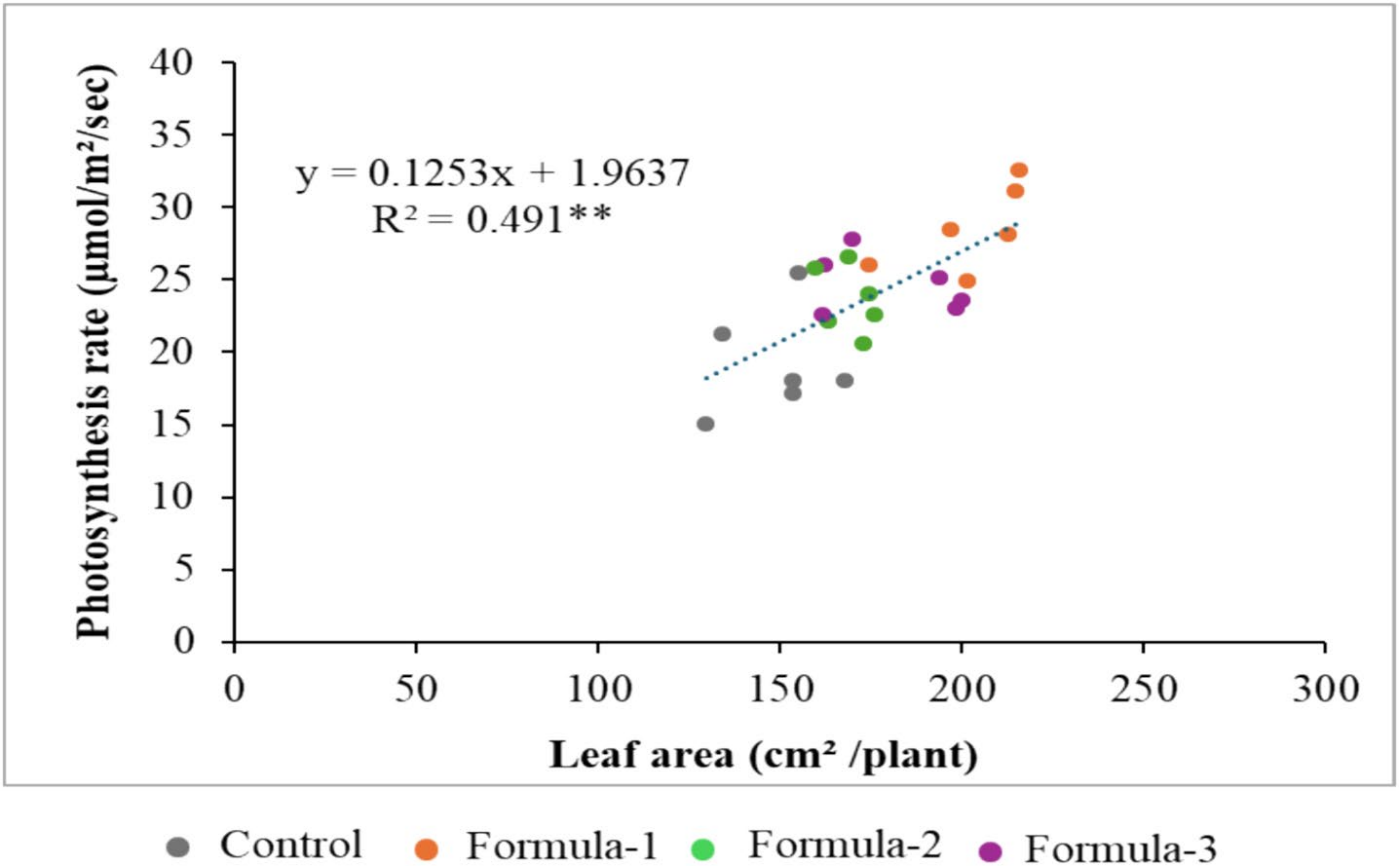
The co-relation between total leaf area per plant at 20 DAP and the rate of photosynthesis during fruit development stage under different NSC. ** indicates significance at *P* < 0.01.

The plant increased in height with low concentrations of nutrient solution compared to control and higher concentrations (Table 4). However, the plants had the lowest shoot dry weight. The formula-3 was found to have the highest fresh weight (2600 kg) and dry weight (294.31 g) compared to other treatments (Table 4) at the end of cultivation. Female flowers were counted from flowering time to the next 20 days, and it was observed that Formula-1 yielded the highest (25) with significant differences with other treatments, while control, Formula-2 and Formula-3 yielded 20, 20 and 19 respectively (Table 4). In the case of leaf area (cm^2^/plant) the highest value was 209.88 cm^2^ was found in Formula-1 while in Control, Formula-2 and Formula-3 the values were 156.07, 158.52 and 186.34 cm^2^/plant respectively (Table 4). Positive correlations were observed between leaf area and photosynthesis rate (Fig. 3). The stem diameter was found to be increased with the decreased NSCs in Formula-1 (Table 4).

### Effects of NSCs on the fruit yield and quality

**Fig. 4:**
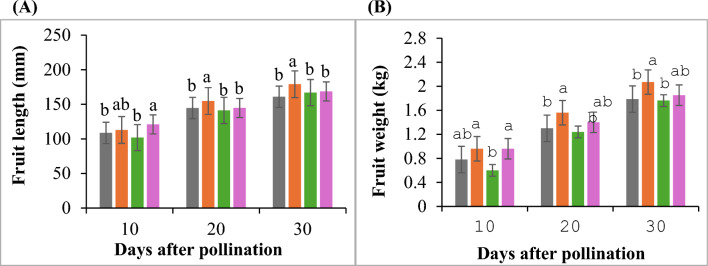
Fruit length (**A**) and fruit weight (**B**) at 10, 20 and 30 Days after pollination under different NSCs. Different letters indicate significance difference (*p* < 0.05; Turkey’s HSD test). Values are the mean ± SE, n = 10.

Plants subjected to different NSCs showed significant differences in fruit lengths and weights (Fig. 3). The fruit length was measured from fruit set time to harvesting time at 10-day intervals. The greatest length was found in Formula-1 which produced bigger fruits; the value was 112.85, 154.78 and 178.98 mm at 10, 20 and 30 DAP respectively (Fig. 4).

In control, Formula-2 and Formula-3 the fruit length was 108.62, 101.79 and 120.9 mm (10DAP); 144.58, 141.12 and 144.63 mm (20DAP); 160.84, 166.82 and 168.56 mm (30DAP) (Fig. 4). The average value of Fruit weights (kg) in Formula-1 at 10, 20 and 30 DAP were 0.96, 1.56 and 2.07 kg which was heavier fruit than for other treatments (Fig. 4). The second highest value was found in Formula-3 at 10, 20 and 30 DAP (0.90, 1.4 and 1.85 kg) while in control and Formula-2 the values were (0.78, 1.3 and 1.78 kg) and (0.6, 1.24 and 1.76 kg) at 10, 20 and 30 DAP respectively (Fig. 4). The yield increases 14.8% in Formula-1 and 106.14% in Formula-3 compared to control (Fig. 4). The percentage of sweetness (°Brix), Formula-1 showed the highest (12.00%) and the lowest was Formula-2 (10.20%) value while the control and Formula-3 were statistically similar with Formula-1 and Formula-2 and the value was 11.50% and 11.10% (Table 5). In addition, fruits from Formula-1 treated plants produce thicker flesh (154.26 mm) compared to Control (135.88 mm), Formula-2 (126.61) and Formula-3 (142.24 mm) and showed highly significant differences among each other (Table 5).

**Table 5:** Different characteristics of fruit with flesh color of hydroponically grown watermelon in vertical farming system inside greenhouse under 4 different treatments.

Treatments	TSS (°Brix)	Acid content (%)	Flesh thickness (mm)	Peel thickness (mm)	PH	Color		
						L*	a*	b*
Control	11.50ab	1.20b	135.88b	3.97c	5.5	27.49	23.35a	12.79b
Formula-1	12.00a	1.07c	154.26a	1.56a	5.64	29.14	24.59a	10.42b
Formula-2	10.20b	1.59a	126.61c	5.351d	5.72	28.55	13.16c	11.37b
Formula-3	11.10ab	1.23b	142.24b	2.01b	5.65	27.56	20.51b	30.33a
F-test	**	**	**	**	ns	ns	**	**
C.V (%)	5.49	21.21	8.79	8.70	21.21	10.12	15.22	22.30

Different letters indicate significance difference (*p* < 0.05; Turkey’s HSD test, *n* = 5) while ns indicate non-significant.

L* indicates lightness.

a* indicates green to red.

b* blue to yellow.

The peel thickness was also found to have significant differences among the treatments. The lower peel thickness was found in Formula-1 which indicated the faster mature compared to other treatments and the value was 1.56 mm, while the highest value 5.35 mm was found in Formula-2. In control and Formula-3 this value was 3.97 mm and 2.01 mm respectively (Table 5). In the case of juice pH, the value was (5.5–5.72) and showed non-significant differences among each other (Table 5). The high acid content was found in Formula-2 (1.59%) and low was in Formula-1 (1.07%) while control and Formula-3 showed no significant differences, and the value was 1.20% and 1.23% respectively. Fruit flesh color coordinates L* were found non significance differences among the treatments and range from 27.49 to 29.14. Significant differences were observed for a* and b* among the treatments and the value was a* (23.35, 24.59, 13.16 and 20.51) and b* (12.79, 10.42, 11.37 and 30.33) in control, Formula-1, Formula-2 and Formula-3 respectively (Table 5).

## Discussion

NSCs as a tool to improve hydroponic sustainability, physiological response, growth, yield and quality of watermelon. The low concentration of NSC, formulated based on the actual requirements of watermelon plants, significantly enhances yield, physiological responses, and quality traits. Formula-1, which contains a low concentration of NSC, demonstrated superior chlorophyll content, photosynthetic rate, stomatal conductance, and transpiration rate compared to all other formulations, which are key determinants of plant growth and productivity^27^ (Table 3). This result indicates that the moderate nutritional stress induced by low NSC enhances nutrient use efficiency, thereby strengthening physiological processes. Previous studies have shown that an increase in NSC levels often negatively affects the growth and photosynthetic capacity of many plants, such as tomatoes, lettuce, and potatoes^14,15,17,28^. Additionally^12^, reported that low NSC influences physiological processes by improving cell division rate and photosynthetic rate, ultimately promoting the growth of sweet potatoes. Apudo et al.^29^ also reported that low NSC exhibited a higher CO2 assimilation rate compared to high NSC. Therefore, the growth of watermelon cultivated with low NSC (Formular-1), which exhibited superior chlorophyll content, photosynthetic rate, stomatal conductance, and transpiration rate, was significantly promoted (Table 4). In particular, the increase in leaf area of watermelons grown with Formula-1 is thought to have facilitated improved light capture and gas exchange, contributing to enhanced physiological activity and yield. Interestingly, low NSC not only led to an increase in leaf area but also enhanced the photosynthetic rate. On the other hand, in Formula-2 and Formula-3 with high nutrient concentrations, osmotic stress likely occurred, impairing stomatal function and potentially reducing overall photosynthetic efficiency. While an increase in NSC resulted in the highest biomass production (Table 4), compared to low NSC (Formula-1), the fruits were smaller (Fig. 3), had lower sugar content (Table 5), and overall yield was also lower (Table 4). These results highlight the importance of balanced nutrient management, as high NSC in Formula-2 and Formula-3 made essential nutrients available in excess without leading to corresponding benefits. As quality indicators of watermelon fruit, juice sugar content, acidity, flesh thickness, and flesh color were examined (Table 5). Fruits produced with Formula-1 (low NSC) had higher sugar content and a redder flesh. It is known that the redder the flesh, the higher the lycopene content. Furthermore, Formula-1 produced fruit with flesh that was 14%, 12%, and 10% thicker than the control, Formula-2, and Formula-3, respectively. However, the peel of a fruit grown with Formula-1 is the thinnest, indicating early maturation. The decrease in acidity observed in fruits produced from plants treated with Formula-1 indicates faster organic acid metabolism during ripening, which implies faster maturation and post-ripening^30^. Tomiyama et al.^31^ observed that high NSC negatively affects watermelon fruit quality. Based on the above, it can be concluded that low concentrations of NSC have a positive impact on the growth and yield of hydroponically grown watermelons.

In this study we observed Formula-1 (low NSC) performs best among other treatments. Why Formula-1 was the best compared to other Treatments this might be due to the optimization of nutrient solutions that matched the plant’s requirements and increased nutrient utilization efficiency^32^ and enhance physiological performance^20^. Formula-1 was prepared by considering low NSC with elevated rate of N (NO3, NH4) and PO4 compared to other macro and micronutrients used in this formula. The reason we elevated the N and P dose is that our preliminary research report showed that watermelon prefer N and P. (Fig. 2). The elevated rate of N and P with low NSC concentration in Formula-1 performed best compared to other treatments when considering growth, physiological responses, yield and quality. Rolbiecki et al.^33^ reported the performance of watermelon can be enhanced when nitrogen is applied at a higher concentration with overall low NSCs through drip irrigation. In contrast, higher NSC in Formula-2 and Formula-3 ensured surplus availability of essential nutrients, but did not match the watermelon plant requirements and decreased the efficiency of nutrient solutions. A similar finding was found by Soufi et al.^34^. In addition, this experiment was conducted in the summer season and the summer is very hot in Japan. High temperatures within the greenhouse caused rapid drying of the coco bags, leading to plant stress when water was insufficient. In hydroponic watermelon cultivation, the nutrients that were provided by the dripping system were not enough during hot weather and frequently dried inside coco bags. To mitigate this, we connected nutrient solutions via a pump system and the pump was turned on for a long time. Due to the large amount of nutrients supply with high concentrations, we think it’s led to an excess of nutrients in the coco bags, leading to less absorption by the plants. Notably, plants exposed to low NSC absorbed more nutrients than those subjected to high NSC, consistent with findings by Oztekin et al. [46,47]. However, plant uptake of the nutrient as a certain level after that plant did not uptake nutrients^35^ shows that if we applied more nutrients in that time, then excessive nutrient application could inhibit plant growth. For example, high nitrogen levels in hydroponic solutions can lead to excessive vegetative growth at the expense of fruit yield in tomato plants^36^. Similarly, high fertigation frequency can lead to nutrient imbalances, although it can also enhance nutrient uptake under certain conditions^37^. Over-application of nutrients can result in toxicity, which manifests as physiological changes in plants^38^ leading to reduced plant growth such as reduction of leaf area, chlorophyll content and photosynthesis decrease as well as overall plant growth also decreased^39^. Finally, fruit growth becomes slow and produces small and low-quality fruits compared to Formula-1 (low NSC). As a result, overall yield decreased, and fertilizer loss and cost of production increased. Thus, the balanced N and P level with optimized NSC in Formula-1 assured the actual nutrient requirements for watermelon plants and emphasizes the precious nutrients management for better responses.

## Conclusion

In this study, the low NSC in Formula-1 was found to improve the physiological response, growth, yield and quality of hydroponically grown watermelons. Chlorophyll content, photosynthesis rate (Pn), stomatal conductance (Gs) and transpiration rate (E) were found to be the maximum under low NSC which confirms the potential physiological response under low NSC. It was also evidence that high NSC has a negative effect on plant physiological process and the yield of watermelon. Hence Formula-1 could be utilized successfully to optimize the NSC for enhancing the growth, yield, and quality of watermelon. The present study suggested that, by tailoring nutrient availability to the specific demands of watermelon plants, growers can achieve higher productivity while maintaining superior fruit characteristics. A more precise adjustment of the NSC increases the yield of watermelons.

## Data Availability

The data that supports the findings of this study are available on request from the corresponding author.
